# Insecticidal Activity of Compounds of Plant Origin on *Mahanarva spectabilis* (Hemiptera: Cercopidae)

**DOI:** 10.3390/insects10100360

**Published:** 2019-10-19

**Authors:** Marcelle L. Dias, Alexander M. Auad, Milena C. Magno, Tiago T. Resende, Marcy G. Fonseca, Sandra E. B. Silva

**Affiliations:** 1 Department of Behavior and Animal Biology, Federal University of Juiz de Fora, Juiz de Fora 36036900, Minas Gerais, Brazil; marcelle.leandrodias@gmail.com; 2 Laboratory of Entomology, Embrapa Dairy Cattle, Juiz de Fora 36038330, Minas Gerais, Brazil; mimaagno@hotmail.com (M.C.M.); tiago.resende@embrapa.br (T.T.R.); marcyfonseca@gmail.com (M.G.F.); 3 Department of Entomology, Federal University of Lavras, Lavras 37200000, Minas Gerais, Brazil; sandraelisa.bio@gmail.com

**Keywords:** spittlebug, IPM, botanical insecticide, biopesticides, main compounds

## Abstract

The damage caused by spittlebugs varies according to the species of grass, and the losses can reach alarming levels. Measures for population control are currently restricted to the use of resistant grasses and the diversification of pastures. Therefore, alternative control measures are necessary, such as the use of botanical insecticides. The aim of this study was to evaluate the insecticidal activities of thymol, carvacrol, eugenol, cinnamaldehyde, and trans-anethole on *Mahanarva spectabilis* eggs, nymphs, and adults under laboratory conditions. In the egg tests, treatments with eugenol, carvacrol, and thymol showed the highest mortalities, presenting efficiencies higher than 85% after 48 h of application. In the nymph tests, the treatments with thymol and carvacrol at 2.5% and eugenol at 2.0% and 2.5% showed intermediate efficiencies, with values above 61%. The highest mortality was observed in the treatment with trans-anethole at 2.5%, with an efficiency of 95%. In the tests with adults, only treatment with trans-anethole at 2.5% obtained an efficiency reaching 90%; in the other treatments, the efficiency did not exceed 51%. These results showed that, at these concentrations, trans-anethole presents a high rate of insecticidal activity on *M. spectabilis* nymphs and adults and, therefore, is recommended as a potential natural insecticide for the control of this pest.

## 1. Introduction

Spittlebugs are the main pests associated with forage grasses in the tropical Americas [1]. The damage caused by spittlebugs varies for each species of grass, and economic losses can reach alarming figures depending on the region, climatic conditions, and management [2]. The global losses caused by these insects are estimated to range from US$840 million to US$2.1 billion per year [3]. *Mahanarva spectabilis* (Distant, 1909) (Hemiptera: Cercopidae) is considered a limiting pest in the production of forage grasses such as elephant grass (*Pennisetum purpureum*, Schumacher) [4]. Although the nymphs of this species cause considerable damage leading to a water imbalance in the plants, the adults are responsible for greater plant losses because they suck the sap and inject a toxin that initiates the yellowing and drying of the forage [5,6]. In addition, this toxin makes pastures unpalatable and hinders the feeding of cattle [7].

The best method to control spittlebugs would be the diversification of pastures and the use of resistant grasses [8]. Although the inclusion of resistant grasses is a promising technique for reducing the damage caused by spittlebugs, there is a long time between the discovery of resistant forage and the release of a cultivar [9]. The chemical control of this pest is considered economically unviable because pasture is considered a low-value crop per unit area and, in addition, the use of insecticides leads to the accumulation of residues [10,11]. Furthermore, these insecticides can decimate entire populations of nontarget organisms [12]. Therefore, alternative control measures are necessary, such as the use of botanical insecticides, which are attractive alternatives for pest management compared with synthetic chemical insecticides because they present little threat to the environment and human health [13,14,15,16]. Natural products are generally efficient, low cost, and less harmful than synthetic products to nontarget organisms, and because of their biodegradability, they are ecologically appropriate [17,18]. Both commercially available formulations and rudimentary essential oils have shown promise in pest control for crops [19]. Recent research has shown a growing interest in the bioactive effects of essential oils and their derivatives on insects [20,21,22,23].

Pure compounds and/or their essential oils thymol, carvacrol, eugenol, trans-anethole, and cinnamaldehyde have already been tested against *Trichoplusia ni* (Hübner, 1803) [24], *Pochazia shantungensis* (Chou and Lu) [25], *Callosobruchus maculatus* (Fabricius, 1775) [26], *Myzus persicae* (Sulzer, 1776), and *Aedes aegypti* (Linnaeus, 1762) [27,28]. Neem-based formulations were also tested against *Mahanarva fimbriolata* (Stal, 1854) [29]. Among the various mechanisms of action of these compounds are the inhibition of acetylcholinesterase and neurotoxic effects involving actions on the receptors of gamma-aminobutyric acid (GABA) and octopamine [30].

Thus, the objective of the present study was to evaluate the insecticidal activities of the monoterpenes thymol and carvacrol, the phenylpropanoid eugenol, the flavonoid trans-anethole, and the ether cinnamaldehyde against the eggs, nymphs, and adults of *M. spectabilis*. 

## 2. Materials and Methods

### 2.1. Acquisition and Maintenance of Insects and Plants

To obtain eggs, *M. spectabilis* adults were collected from the experimental field of Embrapa Dairy Cattle in Coronel Pacheco, MG, Brazil (21°33′22″ S latitude, 43°6′15″ W longitude, at a height above sea level of 414 m). These insects were taken to the Entomology Laboratory of Embrapa Dairy Cattle in Juiz de Fora, MG, Brazil, where they were kept in acrylic cages (30 × 30 × 60 cm) that contained elephant grass plants (*P. purpureum* cv. Roxo de Botucatu) for feeding at a temperature of 25 ± 2 °C. The base of each cage was wrapped with gauze moistened with distilled water, which served as a substrate for oviposition; the wrapped eggs were then placed under a set of sieves and subjected to running water, and the eggs were retained in the finest sieves (mesh opening 400). Then, 300 eggs were grouped in Petri dishes (9 cm in diameter) lined with filter paper and were placed in an air-conditioned chamber, maintained at 25 ± 2 °C with 70% ± 10% relative humidity (RH) and a photoperiod of 12:12 h (L:D) until they reached the S4 development stage, which is characterized by two red spots on each side of the operculum; the operculum corresponds to the eyes, and the red spots represent the nymph’s abdominal pigments [31]. The filter papers were moistened daily, and the development of the eggs was observed. Eggs were used at the S4 stage of development to ensure that the lack of hatching during the tests was caused by the insecticidal substance and not the diapause period of the eggs, which were retained at embryonic stage S2 for approximately 200 days.

To obtain nymphs and adults for bioassays, fourth- and fifth-instar nymphs were collected from the experimental field and sent to the laboratory, where they were placed on elephant grass plants with roots exposed for feeding. Adults were also collected from the field and taken to the laboratory, where they were kept in elephant grass pots covered with voile to prevent the insects from escaping until they were used in the experiment. Prior to bioassays, nymphs and adults collected from the field were conditioned in the laboratory and kept under controlled conditions for 24 h to adapt to the laboratory environment.

Elephant grass (*P. purpureum*) plants were used in 10 cm (single-node) stakes, propagated in plastic pots (500 mL) containing substrate (soil/fertilizer in the proportion 1:1). Seedlings were collected from the experimental field of Embrapa Dairy Cattle in Coronel Pacheco. The seedlings were kept in a greenhouse and irrigated daily until they were used in the experiments (60 days).

### 2.2. Reagents

All compounds were purchased in standard chemical form from Sigma-Aldrich^® ^(Saint Louis, MO, USA). Thymol crystals, eugenol, trans-anethole, and dimethyl sulfoxide (DMSO) were obtained at analytical purity of >99%, and carvacrol and cinnamaldehyde were obtained at purities of 98% and ≥95%, respectively. All solutions were placed in an ultrasonic bath, model Elma E 60 H (Elma Ultrasonic System^®^, Singen, Germany), for approximately 5–7 min, and the thymol solutions were heated to 40 °C for complete emulsion of the crystals.

### 2.3. Evaluation of Insecticidal Activity

In the bioassay of the susceptibility of eggs to botanical compounds, 15 eggs were grouped in Petri dishes (5 cm) lined with filter paper, and each egg received an application of 10 μL (Micropipette V3-PLUS 0.5–10 μL) of solution at concentrations of 0.5%, 1.0%, 1.5%, 2.0%, and 2.5%; these solutions were prepared using 1% DMSO as a solvent or a DMSO control. Each concentration of each compound was replicated 10 times, and the plates of each replicate were maintained in a phytotron-type controlled environment (2.5 × 2.20 × 2.80 m) at 25 ± 2 °C with a photoperiod of 12:12 h (L:D) and RH of 70% ± 10%. The ovicidal activity of each compound was evaluated in a stereomicroscope after 24 and 48 h of application, and the eggs that darkened in color, indicating embryonic death, were considered unviable.

In the tests with nymphs and adults, 10 μL of solution was applied to the dorsal region of each insect at the same concentrations mentioned above, and the insects were then assembled into groups of 10 in plastic pots (500 mL) that contained elephant grass plants. Each concentration of each compound was replicated 10 times, and the pots of each replicate were maintained in a phytotron-type controlled environment. The potted plants containing the nymphs had the roots exposed to jets of running water to facilitate feeding. The pots were wrapped in voile secured by elastic at the base of the leaves to prevent the nymphs from escaping. For the test with adults, a cage accommodating the leaves was adapted to avoid insect escape. The pots were then transferred to an air-conditioned chamber maintained at 25 ± 2 °C with a 12 h photophase and RH of 70% ± 10%. Then, the number of survivors was counted 24 and 48 h after the treatments. The insects were considered dead when they presented evidence of paralysis, tipping, and immobility when touched by the bristles of a fillet-type brush after 60 s under these conditions.

### 2.4. Statistical Analysis

According to the National Health Surveillance Agency (ANVISA) [32], tests with insecticides and related products are considered satisfactory when the mortality obtained in the positive control reaches an average value of 90% (±10%) in relation to the control. In the present research, we considered treatments to be satisfactory if they were efficient according to ANVISA. The experimental design consisted of randomized blocks composed of 25 treatments and a control with 10 replicates. In each repetition, 15 eggs or 10 nymphs or adults were used. The control efficiency of the treatments was calculated using the Abbott formula [33]. The data were transformed by the square root of (x + 0.5) for the analysis of variance (ANOVA) of repeated measurements, and the means were compared by the Scott Knott test (*p* < 0.05). The analyses were performed using the free software RStudio with R version 3.5.1 (packages: ScottKnott [v1.2-7]) [34,35]. 

## 3. Results

**Figure 1:**
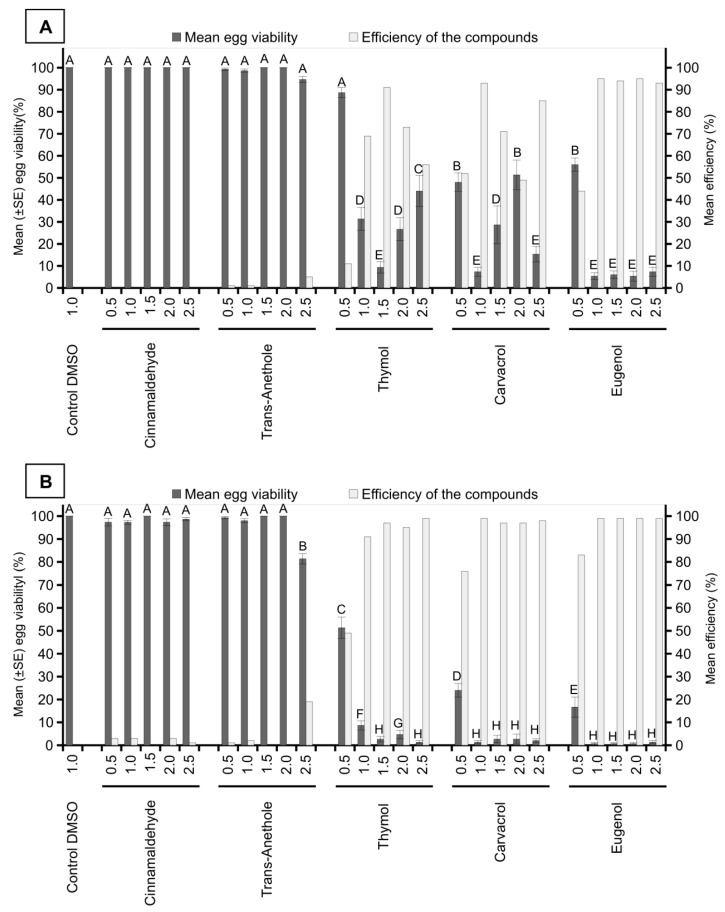
Insecticidal activity of compounds of plant origin (concentrations of 0.5%, 1.0%, 1.5%, 2.0%, and 2.5%) against *Mahanarva spectabilis* eggs after 24 (**A**) and 48 h (**B**) of application. The control efficiency of the treatments was calculated using the Abbott formula. Different letters in the columns represent significant differences between the treatments by the Scott Knott test (*p *< 0.05).

In the evaluation performed 24 h after the application of the treatments, the number of viable eggs was significantly lower at concentrations of 1.0%, 1.5%, 2.0%, and 2.5% in the thymol treatments and at all concentrations in the treatments with carvacrol and eugenol when compared with the control; the same results were not obtained for the other treatments (F = 62.37; *df *= 25; *p* < 0.0001). In the present research, the treatments with thymol at a concentration of 1.5%; carvacrol at concentrations of 1.0% and 2.5%; and eugenol at concentrations of 1.0%, 1.5%, 2.0%, and 2.5% showed the best results, presenting control efficiencies higher than 85%. In addition, thymol treatments at concentrations of 1.0% and 2.0% and carvacrol at 1.5% showed intermediate control efficiencies above 69%. Efficiencies below 60% were found for thymol at 0.5% and 2.5%, carvacrol at 0.5% and 2.0%, and eugenol at 0.5%, whereas zero efficiency was observed for all concentrations of cinnamaldehyde, and efficiencies below 5% were observed for all concentrations of trans-anethole (Figure 1A).

At 48 h after application, the number of viable eggs was significantly lower in the treatments with thymol, carvacrol, and eugenol at all concentrations and in that with trans-anethole at 2.5% when compared with the control treatment (F = 266.90; *df *= 25; *p* < 0.0001). The treatments with thymol, carvacrol, and eugenol at concentrations of 1.0%, 1.5%, 2.0%, and 2.5% presented the best results in relation to the other treatments, showing control efficiency values greater than 91% and reaching 99%. In addition, the carvacrol and eugenol treatments at the lowest dose (0.5%) presented control efficiencies of 76% and 83%, respectively. The treatments with cinnamaldehyde and trans-anethole at different concentrations showed control efficiencies below 5% and 20%, respectively (Figure 1B).

**Figure 2:**
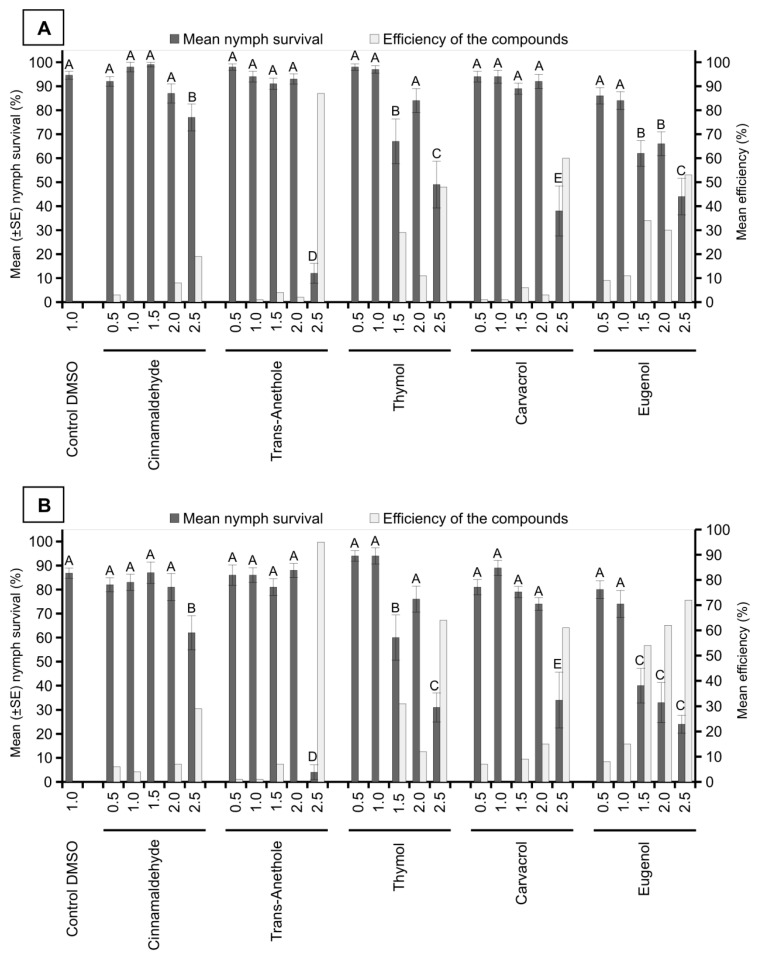
Insecticidal activity of compounds of plant origin (concentrations of 0.5%, 1.0%, 1.5%, 2.0%, and 2.5%) against *M. spectabilis* nymphs after 24 (**A**) and 48 h (**B**) of application. The control efficiency of the treatments was calculated using the Abbott formula. Different letters in the columns represent significant differences between the treatments by the Scott Knott test (*p* < 0.05).

In the first evaluation, performed 24 h after the treatments, the number of surviving nymphs was significantly lower in the thymol treatments at 1.5% and 2.5%; carvacrol at 2.5%; eugenol at 1.5%, 2.0%, and 2.5%; and trans-anethole at 2.5% when compared with that in the control treatment (F = 20.95; *df* = 25; *p* < 0.0001). The best results were observed in the treatments with carvacrol and trans-anethole at a concentration of 2.5%, with control efficiency values of 60% and 87%, respectively. In the other treatments, the control efficiency was lower than 60% (Figure 2A).

In the evaluation after 48 h of application, treatments with thymol at 1.5% and 2.5%; carvacrol at 2.5%; eugenol at 1.5%, 2.0%, and 2.5%; and cinnamaldehyde and trans-anethole at 2.5% presented significantly fewer surviving nymphs when compared with the control treatment (F = 24.14; *df *= 25; *p* < 0.0001). In the treatments with thymol and carvacrol at 2.5% and eugenol at 2.0% and 2.5%, intermediate control efficiency was observed, with values above 61%. The best result was observed in the treatment with trans-anethole at 2.5%, with a control efficiency of 95%. In the other treatments, the control efficiency was below 60% (Figure 2B).

**Figure 3:**
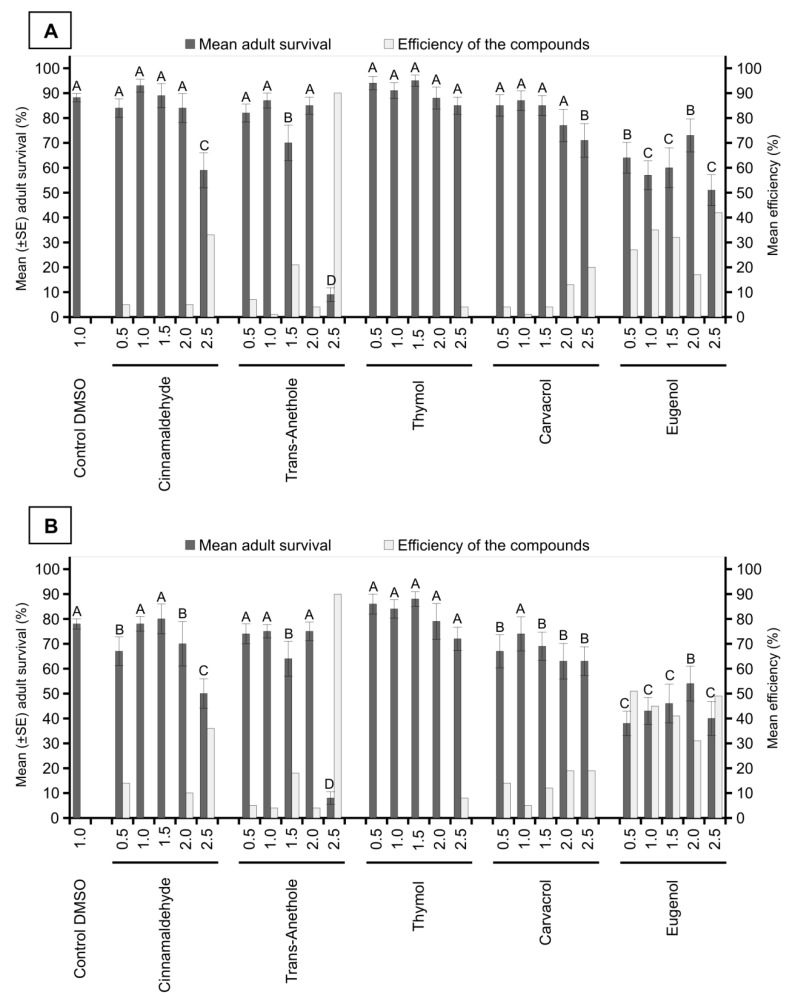
Insecticidal activity of compounds of plant origin (concentrations of 0.5%, 1.0%, 1.5%, 2.0%, and 2.5%) against *M. spectabilis* adults after 24 (**A**) and 48 h (**B**) of application. The control efficiency of the treatments was calculated using the Abbott formula. Different letters in the columns represent significant differences between the treatments by the Scott Knott test (*p* < 0.05).

In the evaluation performed 24 h after application, the number of surviving adults was significantly lower in the treatments with carvacrol at 2.5%, eugenol at all concentrations, cinnamaldehyde at 2.5%, and trans-anethole at 1.5% and 2.5% when compared with that in the control treatment (F = 18.39; *df* = 25; *p* < 0.0001). Only the treatment with trans-anethole at 2.5% showed a control efficiency of 90%, whereas in the other treatments, the efficiency ratio did not exceed 42% (Figure 3A).

In the evaluation performed 48 h after application, the number of surviving adults was significantly lower in the carvacrol treatments at 0.5%, 1.5%, 2.0%, and 2.5%; eugenol at all concentrations; cinnamaldehyde at 0.5%, 2.0%, and 2.5%; and trans-anethole at 1.5% and 2.5% when compared with that in the control treatment (F = 12.73; *df* = 25; *p* < 0.0001). Once again, only in the treatment with trans-anethole at a concentration of 2.5% did the control efficiency reach 90%, whereas in the other treatments, the efficiency ratio did not exceed 51% (Figure 3B).

## 4. Discussion

Compounds from essential oils are attractive alternatives for the management of this pest because they are safer than synthetic insecticides both for the environment and human health [36].

The results of the experiments showed that an evaluation performed 24 h after application was not able to determine the unviability rate of eggs, and even with increased concentrations, there was no corresponding increase in mortality. This may have occurred due to the necessity of a longer time for the product to act on the eggs, which suggests that evaluations of bioinsecticide tests should be performed 48 h after their application. At 48 h after application, treatments with thymol, carvacrol, and eugenol showed rates varying between 91% and 99% for *M. spectabilis* egg unviability. This result can be explained by the presence of structures described as numerous small pores present in the exochorion of eggs of the species *M. fimbriolata* [37]. These pores are probably responsible for the oxygenation of the inner structures of the eggs and can also act as facilitators for the introduction of toxic materials into the egg membrane [38].

The ovicidal effects of these monoterpenes against other entomological pests, such as the effect of thymol and carvacrol on the eggs of *Rhodnius prolixus* (Stal, 1859) (Hemiptera: Reduviidae) and the effect of eugenol on eggs of *Bradysia procera* (Winnertz, 1868) (Diptera: Sciaridae), have already been mentioned in the literature [39,40]. The modes of action of thymol and its isomer carvacrol, such as thymol’s ability to block GABA and/or octopaminergic insect systems [41,42,43] and the inhibitory effects of acetylcholinesterase exhibited by carvacrol [44] on domestic flies, ticks, and cockroaches, have also been reported in the literature. Although the neurotoxic action of these monoterpenes is widely described in the literature, the toxicity of these compounds becomes more visible when the nervous system of the insect embryo is developing [39].

The efficiency rates of eugenol on eggs varied between 83% and 99% egg unviability in both evaluations. The ovicidal effect of this compound was evaluated in tests in which eugenol was applied to the eggs of *Sitophilus granarius* (Linnaeus, 1758) and *Sitophilus zeamais* (Motschulsky, 1855) (Coleoptera: Curculionidae); these studies concluded that eugenol completely inhibits egg hatching in these insects [45]. Eugenol was also reported as a neuroinsecticide against the ant *Camponotus pennsylvanicus *(De Geer, 1773) (Hymenoptera: Formicidae), and the octopaminergic system acts as a mediator of its insecticidal activity [46].

The compounds used in this study were more effective against eggs than against the nymphs of *M. spectabilis*. Similar results were found in tests comparing the effectiveness of some insecticides, including *Quassia amara* (Linnaeus) (Simaroubaceae), NeemAzal, and other botanical insecticides, against *Aleyrodes proletella* (Linnaeus, 1758) (Hemiptera: Aleyrodidae); this difference was attributed to the serous layer that protects the nymphs [47]. In our research, we suggest that, in addition to the presence of pores in the egg shells, this difference may have occurred due to the froth secreted by the nymphs of spittlebugs. It is proposed that the main functions of this foam are to confer protection against predation and to form a microhabitat that avoids high-temperature dryness and helps the thermoregulation of nymphs [48,49,50]. However, there may be a similarity between the foam produced by the spittlebugs and the foam that snails secrete during a physical attack or exposure to chemicals [51]. Thus, we suggest that the production of froth by the nymphs soon after the application of the treatments functions to partially eliminate the irritant.

At least one concentration of thymol, carvacrol, and eugenol exhibited control efficiency rates above 60% on the nymphs at 48 h. These compounds have already been proved effective on other insects, for example, the potential of eugenol, thymol, and carvacrol on nymphs of *R. prolixus* (Stal, 1859) and *Triatoma infestans *(Klug, 1834) (Hemiptera: Reduviidae), which are vectors of Chagas’ disease [52]. Likewise, the insecticidal properties of thymol and carvacrol, derived from the essential oil of *Thymus vulgaris* (Linnaeus) (Lamiaceae), were proved on *P. shantungensis* nymphs [25]. In the nymph tests, trans-anethole obtained efficiency rates between 87% and 95%, superior to those observed in nymphs of *Trialeurodes vaporariorum* (Westwood, 1856) (Hemiptera: Aleyrodidae), in which the compound showed 50% interference in nymphal growth [53]. Studies have shown the toxic effects of phenylpropanoid trans-anethole on other pest insects, such as *Spodoptera frugiperda* (Smith, 1797) (Lepidoptera: Noctuidae) [54] and *Tribolium castaneum* (Herbst, 1797) (Coleoptera: Tenebrionidae) [55]. Among the effects of trans-anethole, there are reports of changes in the biological parameters of insects, such as the inhibition of acetylcholinesterase [56], which may explain its efficacy in the nymphs of this study.

The least efficient compound against *M. spectabilis* nymphs was cinnamaldehyde, which was in contrast to the results found in tests of the insecticidal activity of cinnamon essential oils, their constituents, and the analogues of (E)-cinnamaldehyde on *Metcalfa pruinosa* nymphs and adults (Say, 1830) (Hemiptera: Flatidae) [57]. This difference in toxicity may have occurred due to the particularities of the target species of each study, such as physiology and resistance mechanisms.

In the tests with adults, the efficiency rates of the treatments were much lower than those obtained in the tests with nymphs. We can infer that this difference may have occurred due to the adult integument being more chitinized, acting as a physical barrier to the absorption of the applied compounds, a fact already mentioned in the literature to explain the absence of adult infection of *M. spectabilis* by entomopathogenic nematodes [58]. However, trans-anethole presented an efficiency rate of 90% when applied at the highest concentration to the adults of *M. spectabilis*. This efficiency can be explained by the double bond of the propenyl group present in trans-anethole, suggested in toxicity tests on *Callosobruchus chinensis* (Linnaeus, 1758) (Coleoptera: Bruchidae) to be responsible for the high insecticidal activity of this compound [59]. Eugenol showed an efficiency rate below 60% in adults, unlike the results found in adults of *M. pruinosa*, against which eugenol was mentioned as the most toxic compound [57]. In our evaluations, thymol presented a significantly lower activity than that of carvacrol in adults, as found in tests of the activity of these compounds on *Culex quinquefasciatus* larvae and pupae (Say, 1823) (Diptera: Culicidae) [60]. It is worth noting that the only structural difference between these two compounds is the position of the hydroxyl group on the benzene ring relative to the larger aliphatic chain and may be related to this difference in activity [60]. However, it is important to note that the efficacy of thymol is well reported for several insect species, such as *Anopheles stephensi* (Liston, 1901) (Diptera: Culicidae) [61], *S. zeamais* [62], and *Plutella xylostella* (Linnaeus, 1758) (Lepidoptera: Plutellidae) [19].

## 5. Conclusions

This research shows, for the first time, the activity of some major compounds of vegetal origin in the different phases of life of *M. spectabilis*. Eugenol, carvacrol, and thymol were the most efficient compounds in reducing the number of spittlebug eggs. However, control of this insect should focus on decreasing the number of nymphs and adults. For these phases, the trans-anethole activity had control efficiency rates higher than 85%, a value within the recommended insecticide registration range. Thus, taking into account the concentrations tested, this compound is recommended as a potential natural insecticide for the control of *M. spectabilis*.
